# Methodology of stakeholders’ behaviour modelling based on time

**DOI:** 10.1016/j.mex.2021.101570

**Published:** 2021-11-02

**Authors:** Michal Munk, Anna Pilkova, Lubomir Benko, Petra Blazekova, Peter Svec

**Affiliations:** a Constantine the Philosopher University in Nitra, Slovakia; b Comenius University in Bratislava, Slovakia

**Keywords:** Data pre-processing, Web usage mining, Multinomial logit model

## Abstract

The methods presented in this article were created to model and describe the behaviour of the web users of a bank institution web portal. The source dataset is represented by a log file of the commercial bank web server. The analysis is oriented on examining the behaviour of visitors over an extended period (2009-2012). The years 2009-2010 represent the years of the financial crisis, and the years 2011-2012 represent the years after the financial crisis. The following method describes the sequence of steps necessary to pre-process the raw log file and model the web user behaviour using the multinomial logit model. The introduced methods can be used also for other domains in the case of appropriate data preparation.

• Data preparation- data cleaning, user/session identification, path completion, variables determination;
• Data analysis- model definition, parameters estimation, logits estimation, probabilities estimation;
• Results evaluation- comparison of empirical and theoretical values in term of counts, probabilities and logits.

## Graphical abstract

**Figure:**
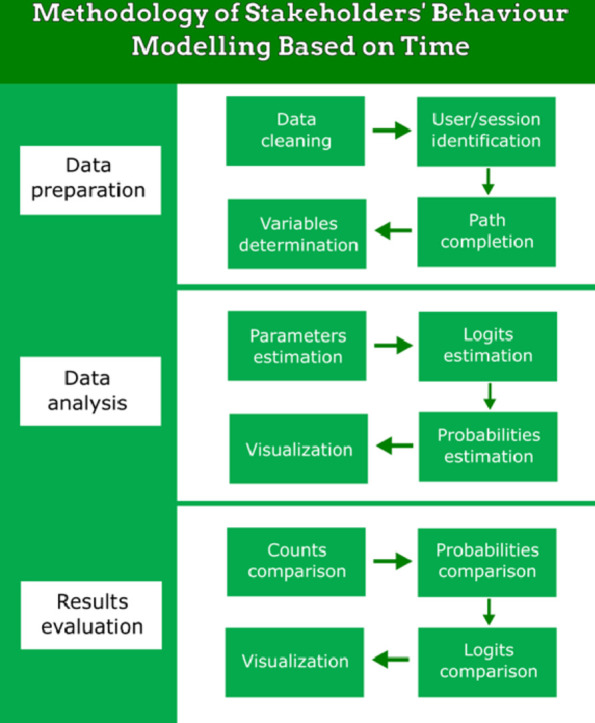

Specification table

**Table:** 

Subject Area	Computer Science
More specific subject area	*Web Usage Mining*
Method name	*Methodology of Stakeholders’ Behaviour Modelling Based on Time*
Name and reference of original method	*Web usage data pre-processing and analysis* [1,2]
Resource availability	*The pre-processed log file is located in Data in Brief*[3]*.*

Methods described in this article were created to analyse and model the behaviour of the visitors of a web portal – bank web portal for a research article [4]. The data source was log files obtained from the webservers and it contained the visitors’ accesses to the web parts of the web portal. A detailed description of the log files is located in Data in Brief [3]. Data related to Pillar 3 were gathered from bank webserver log files [5]. The research methodology was inspired by [6], [7], [8]. The model of bank visitors’ behaviour was created based on a multinomial logit model [9]. The web usage analysis was done based on a sample of 2 071 235 logged accesses that were obtained after data preparation. The investigated categorical dependent variable is a variable *category* that represents a group of web parts that deal with a similar issue. The variable contains these categories: *Pricing List, Reputation, Business Conditions, Pillar3 related, Pillar3 disclosure requirements* and *We support*. The analysis is oriented on examining the behaviour of visitors over an extended period of time (2009-2012). The years 2009-2010 represent the years of financial crisis (variable *crisis=1*). On the other hand, the years 2011-2012 represent the years after the financial crisis (variable *crisis=0*). The time independent variable was chosen as the variable *week* that was created from the date of the access of the visitor. The variable was created based on the standard ISO 8601 and the variable acquires the values 0-53. If the week number equals 0, it means that the given date belongs to the preceding year. The applied methodology is based on [10] and is as follows:

1. Obtaining log files from multiple servers.
2. Data preparation involving the following multiple tasks:
  a. Data cleaning – removing the unnecessary data from the log files (requests for pictures, styles and so on; and also accesses of robots of search engines) which leads to raw data of only accesses to the web portal.
  b. User/session identification – the visitors were identified based on the variables IP address and user agent; and sessions were identified based on the Reference Length method.
  c. Path completion –used to complete the records of the users’ path that the user followed using the Back button in the web browser (these visited pages are not recorded in the log file since they have already been stored on the client side under the previous steps).
  d. Variables determination – the log file contains the variables in a typical Extended Log Format (ELF), so a transformation and variable definition are needed for the user behaviour analysis of the examined web portal. A dependent variable *category* is created, and it represents the web parts of the web portal. In case the web parts have low traffic, it is appropriate to create wider categories based on their relevance to the content [9]. The variable *category* will in the case of the examined web portal of the bank institution contain the following web categories: *Pricing List, Reputation, Business Conditions, Pillar3 related, Pillar3 disclosure requirements,* and *We support*. It is also necessary to identify independent variables- predictors that represent the time variables created from the timestamp of the access to the web category. In case of the weeks of the year, it is the variable *week* that was created based on ISO 8601 and will have values of 0-53. The variable will be 0 in case it is a week that begins in the previous year. The next predictor will be the dummy variable *crisis* that identifies the period of years during a financial crisis and after a financial crisis. Next a dummy variable *internal* was created for the identification of accesses from inside and outside of the organization's network. In this way the behaviour of users accessing from the inside/outside (internal/external access) of the organizations’ network (the variable was created based on the sets of IP addresses) can be analysed.
3. Data analysis follows based on the presumption that the examined data consists of individual accesses to web portal parts:
  a. Model definition - probability distribution of accesses $Y_{ij}$ in time ifor the category j with observations $y_{ij}$, if the count of accesses is given $n_{i}=\sum_{j}y_{ij}$ in time i is multinomial $P\left[Y_{i1}=y_{i1},Y_{i2}=y_{i2},\ldots ,Y_{iJ}=y_{iJ}\right]=\frac{n_{i}!}{y_{i1}!y_{i2}!\ldots y_{iJ}!}\pi_{i1}^{y_{i1}}\pi_{i2}^{y_{i2}}\ldots \pi_{iJ}^{y_{iJ}}$. Since $\sum_{j=1}^{J}\pi_{ij}=1$ it is necessary to estimate J−1 of unknown probabilities. The estimates are calculated using the Maximum Likelihood method. In the logarithmic function of likelihood (without constants) $\sum_{i}\sum_{j=1}^{J}y_{ij}\ln\pi_{ij}$ (1) is denoted a logit transformation $\eta_{ij}=\ln\frac{\pi_{ij}}{\pi_{iJ}}$, where the last category is chosen as the reference category $\eta_{iJ}=0$, and it is assumed that the logits $\eta_{ij}$ are linear functions of the independent variables $\eta_{ij}=\alpha_{j}+x_{i}^{T}\beta_{j}$. Using inverse transformation, it is denoted $\pi_{iJ}=\frac{1}{1+\sum_{j=1}^{J-1}e^{\eta_{ij}}},\pi_{ij}=e^{\eta_{ij}}\pi_{ij},j=1,\;2,\ldots ,\;J-1$, respectively $\pi_{iJ}=\frac{1}{1+\sum_{j=1}^{J-1}e^{\alpha_{j}+x_{i}^{T}\beta_{j}}},\pi_{ij}=\frac{e^{\alpha_{j}+x_{i}^{T}\beta_{j}}}{1+\sum_{j=1}^{J-1}e^{\alpha_{j}+x_{i}^{T}\beta_{j}}},j=1,\;2,\ldots ,J-1$ (2). The logarithm function is a likelihood function with unknown parameters $\alpha_{j}$ and $\beta_{j}$ (j=1,2,…,J−1) after substituting such expressed $\pi_{ij}$ (2) into (1). After determining the model, it is necessary to identify the type of dependence for determining the degree of polynomial and the selection of predictors, including dummy variables.
  b. The estimation of the models’ parameters $\alpha_{j},\beta_{j}$ by maximizing the logarithm of the multinomial likelihood function. The *STATISTICA Generalized Linear/Nonlinear Models* was used to estimate the parameters of individual values*.* The significance of parameters was tested using the Wald test $H0:\;\alpha_{j}=0$, $H0:\;\beta_{kj}=0$, where *k* is the number of predictors. The estimated parameters are used to calculate estimates of logits and from logits can be calculated probabilities of selection of specific categories at a given time.
  c. The estimation of logits $\eta_{ij}$ for all values of independent variables ${\hat{\eta }}_{ij}=a_{j}+x_{i}^{T}b_{j},j=1,\;2,\ldots ,J-1$.
  d. Probability estimation of accesses $\pi_{iJ}$ in time i for reference web category $J\;{\hat{\pi }}_{iJ}=\frac{1}{1+\sum_{j=1}^{J-1}e^{{\hat{\eta }}_{ij}}}$.
  e. Probability estimation of accesses $\pi_{ij}$ in time i for web category  ${\hat{\pi }}_{ij}=e^{{\hat{\eta }}_{ij}}{\hat{\pi }}_{iJ},j=1,\;2,\ldots ,J-1$.
  f. Visualization of the probabilities of web category j in time i, where j=1,2,…,J.
4. Results evaluation:

Based on the assumption that the expected counts ${\hat{y}}_{ij}=n_{i}{\hat{\pi }}_{ij}$ are big enough (they are not zero and no more than 20% from ${\hat{y}}_{ij}$ is less than 5) to compare the actual model with the saturated model that is used to predict the probabilities independently for i=0,1,…,53, then the statistics $G^{2}$ (deviance, likelihood ratio) can be used

$$G^{2}=LR\left(\hat{\pi }\right)=2\left(L\left(p\right)-L\left(\hat{\pi }\right)\right)=2\sum_{i=0}^{53}\sum_{j=1}^{J}y_{ij}\left(\ln p_{ij}-\ln{\hat{\pi }}_{ij}\right)=2\sum_{i=0}^{53}\sum_{j=1}^{J}y_{ij}\ln\frac{p_{ij}}{{\hat{\pi }}_{ij}}=2\sum_{i=0}^{53}\sum_{j=1}^{J}y_{ij}\ln\frac{y_{ij}}{n_{i}{\hat{\pi }}_{ij}}.$$

After the last edit it is following:

$$G^{2}=2\sum_{i=0}^{53}\sum_{j=1}^{J}y_{ij}\ln\frac{y_{ij}}{{\hat{y}}_{ij}}.$$

The hypothesis $H0:\;\pi_{ij}={\hat{\pi }}_{ij}$ can be testes using the LR test. LR test makes it possible to compare the estimates ${\hat{y}}_{ij}$ with $y_{ij}$. The saturated model has 54(J−1) free parameters and the current model k(J−1), then the degrees of freedom *df* are equal (54−k)(J−1). Statistics $G^{2}$ has approximately $\chi^{2}\left(df\right)$ distribution. Pearson statistics can be used to compare the estimates ${\hat{y}}_{ij}$ with $y_{ij}$ either:

$$\chi^{2}=\sum_{i=0}^{53}\sum_{j=1}^{J}r_{ij}^{2},$$

Where $r_{ij}=\frac{y_{ij}-{\hat{y}}_{ij}}{\sqrt{{\hat{y}}_{ij}}}$ is Pearson residue that has also the $\chi^{2}\left(df\right)$ distribution.

In the given application field is often the condition of using the LR test/ Pearson statistics violated. Usually, the examined variable has a considerable number of levels that are web parts of the portal or system (pages, content categories, activities, etc.). This results in the violation of using the LR test/Pearson statistics- the expected counts are not large enough. For this reason are used alternative methods to evaluate the model [9,11] - visualization of empirical and theoretical counts differences, extreme identification, comparison of the distribution of empirical relative counts of accesses and estimated probabilities of the examined web part *j* in time *i*, and empirical and theoretical logit visualization of each web part, except the reference web part.

The created model was evaluated based on the following steps:

a. Empirical counts determination $y_{ij}$.
b. Theoretical counts estimation ${\hat{y}}_{ij}={\hat{\pi }}_{ij}\sum_{j}y_{ij}$.
c. Visualization of differences in the empirical and theoretical counts of accesses $d_{ij}=y_{ij}-{\hat{y}}_{ij}$.
d. Extreme values identification $d_{ij}$, where $d_{ij}>{\bar{d}}_{j}+2s_{j}$ represents an underestimated prediction and $d_{ij}<{\bar{d}}_{j}-2s_{j}$ represents overestimated prediction where $s_{j}$ is standard deviation and ${\bar{d}}_{j}$ is the mean of differences of the category j.
e. Calculation of relative empirical counts of accesses $p_{ij}=\frac{y_{ij}}{\sum_{j}y_{ij}}$.
f. Comparison of the distribution of the relative empirical counts of accesses with the estimated probabilities of the selected web category j in time i. To test the zero hypothesis, dividing the pair differences is symmetric around zero $r_{ij}=p_{ij}-\pi_{ij}$, H0: F(−r)=1−F(r), a Wilcoxon pair test will be used.
g. Calculation of empirical logits $h_{ij}=\ln\left(\frac{p_{ij}}{p_{iJ}}\right),j=1,\;2,\ldots ,J-1$.
h Visualization of empirical and theoretical logits for individual web categories except for the reference one.

## Declaration of Competing Interest

The authors declare that they have no known competing financial interests or personal relationships that could have appeared to influence the work reported in this paper.
